# Lysosomal localization of GLUT8 in the testis – the EXXXLL motif of GLUT8 is sufficient for its intracellular sorting via AP1- and AP2-mediated interaction

**DOI:** 10.1111/j.1742-4658.2009.07089.x

**Published:** 2009-07

**Authors:** Muhammed Kasim Diril, Stefan Schmidt, Michael Krauß, Verena Gawlik, Hans-Georg Joost, Annette Schürmann, Volker Haucke, Robert Augustin

**Affiliations:** 1 Institute of Chemistry and Biochemistry, Department of Membrane Biochemistry, Freie Universität & Charité Universitätsmedizin BerlinTakustrasse 6, Berlin, Germany; 2 Department of Pharmacology, German Institute of Human Nutrition, Potsdam RehbrueckeArthur-Scheunert-Allee 114–116, Nuthetal, Germany

**Keywords:** adaptor proteins, endocytosis, glucose transporter, GLUT8, lysosomes, targeting

## Abstract

The class III sugar transport facilitator GLUT8 co-localizes with the lysosomal protein LAMP1 in heterologous expression systems. GLUT8 carries a [D/E]XXXL[L/I]-type dileucine sorting signal that has been postulated to retain the protein in an endosomal/lysosomal compartment via interactions with clathrin adaptor protein (AP) complexes. However, contradictory findings have been described regarding the subcellular localization of the endogenous GLUT8 and the adaptor proteins that interact with its dileucine motif. Here we demonstrate that endogenous GLUT8 is localized in a late endosomal/lysosomal compartment of spermatocytes and spermatids, and that the adaptor complexes AP1 and AP2, but not AP3 or AP4, interact with its N-terminal intracellular domain (NICD). In addition, fusion of the GLUT8 NICD to the tailless lumenal domain of the IL-2 receptor alpha chain (TAC) protein (interleukin-2 receptor α chain) targeted the protein to intracellular membranes, indicating that its N-terminal dileucine signal is sufficient for endosomal/lysosomal targeting of the transporter. The localization and targeting of GLUT8 show striking similarities to sorting mechanisms reported for lysosomal proteins. Therefore, we suggest a potential role for GLUT8 in the so far unexplored substrate transport across intracellular membranes.

**Structured digital abstract**

- MINT-7035377: *GLUT8* (uniprotkb:Q9JIF3) *physically interacts* (MI:0915) with *AP2* (uniprotkb:P62944) by *pull down* (MI:0096)
- MINT-7035218: *GLUT8* (uniprotkb:Q9JIF3) *physically interacts* (MI:0915) with *AP1* (uniprotkb:O43747) by *pull down* (MI:0096)
- MINT-7035273: *GLUT8* (uniprotkb:Q9JIF3) *physically interacts* (MI:0915) with *AP1* (uniprotkb:P22892) by *pull down* (MI:0096)
- MINT-7035235: *GLUT8* (uniprotkb:Q9JIF3) *physically interacts* (MI:0915) with *AP1* (uniprotkb:Q8R525) by *pull down* (MI:0096)
- MINT-7035360: *GLUT8* (uniprotkb:Q9JIF3) *physically interacts* (MI:0915) with *AP2* (uniprotkb:Q9DBG3) by *pull down* (MI:0096)
- MINT-7035789, MINT-7035807: *lamp1* (uniprotkb:P11438) and *GLUT8* (uniprotkb:Q9JIF3) *colocalize* (MI:0403) by *fluorescence microscopy* (MI:0416)
- MINT-7039929, MINT-7039945: *lamp2* (uniprotkb:P17047) and *GLUT8* (uniprotkb:Q9JIF3) *colocalize* (MI:0403) by *fluorescence microscopy* (MI:0416)

## Introduction

Facilitative hexose transport is mediated by 14 isoforms of the glucose transporter protein family (GLUT) [1,2]. Based on sequence homology, three classes can be distinguished. Class III family members are unique in containing a tyrosine or dileucine motif that is responsible for their intracellular rather than plasma membrane localization [2].

The initial characterization of endogenous GLUT8 in mouse pre-implantation embryos suggested that GLUT8 mediates insulin-stimulated glucose transport in blastocysts [3]. In contrast, translocation of GLUT8 to the plasma membrane in response to insulin or other stimuli was not observed in several other *in vitro* studies [4–7]. With the exception of the myo-inositol transporter HMIT [H(+)-myo-inositol transporter GLUT13], which is recruited from an intracellular pool to the plasma membrane in response to various stimuli [8], no mechanism for translocation of class III family members has been described, therefore questioning their functional significance in mediating hexose transport across the plasma membrane [5,7,9]. The class III family members GLUT6 and GLUT8 were detected in plasma membranes only after mutation of their dileucine motifs to alanines [4,5,10]. Stably overexpressed GLUT8 co-localized with the late endosomal/lysosomal protein LAMP1 [4,7]. This localization is probably mediated by its N-terminal [D/E]EXXXL[L/I] consensus sequence, which represents a late endosomal/lysosomal sorting signal [4].

GLUT8 is mainly expressed in testis and to a lesser extent in brain [11,12]. Contradictory data exist regarding its localization in the tissues in which it is most abundant. GLUT8 has been found to be localized to the acrosomal membrane of mature spermatozoa [13], while another report found that the protein was localized to the acrosome, mid- and endpiece of spermatozoa, as well as in Leydig cells [14]. A third study detected GLUT8 only in differentiating spermatocytes but not in mature spermatozoa [15].

Heterotetrameric adaptor protein (AP) complexes mediate membrane protein sorting in the secretory or endocytotic pathway by recognizing specific signals within the cytoplasmic portion of their respective cargo proteins [16,17]. The various AP complexes (AP1–4) control protein trafficking to and from various compartments [18]. Signals known to interact with AP complexes conform either to tyrosine-based (YXXø) or dileucine-based ([DE]XXXL[LI]) consensus sequences (where X represents any amino acid and Ø is a bulky hydrophobic residue) [16]. For GLUT8, interaction of the dileucine motif with subunits of AP1 and AP2 has been reported on the basis of glutathione *S*-transferase (GST) pulldown assays with recombinant AP subunits [19,20]. However, the findings have been contradictory with regard to localization of the endogenous GLUT8 in testis, the nature of its sorting, and the interaction of its N-terminal dileucine motif with the various AP subunits. The [DE]XXXL[LI] signal of GLUT8 has been shown to bind to the β2-adaptin subunit of AP2 [20], but a second study identified γ/δ1 and α/δ2 hemicomplexes of AP1 and AP2 as the subunits responsible for the interaction [19].

In the present study, we aim to resolve some of these discrepancies in order to (a) identify the subcellular localization of GLUT8 in testis, (b) elucidate the role of APs in GLUT8 sorting, and (c) understand the role of the EXXXLL motif in GLUT8 sorting. The data provide evidence that endogenous GLUT8 co-localizes with the lysosomal proteins LAMP1 and LAMP2 in spermatocytes and spermatids. The EXXXLL motif interacts with AP1 and AP2 but not with AP3 or AP4, and appropriate targeting of GLUT8 is dependent on both AP1 and AP2, while AP3 is not required. Using lumenal domain of the IL-2 receptor alpha chain (TAC) chimeric proteins we demonstrate that the dileucine motif of GLUT8 represents a strong internalization signal that appears to be sufficient to retain the transporter in an endosomal/lysosomal compartment.

## Results

### GLUT8 co-localizes with lysosomal proteins in mouse testis sections

**Fig. 1:**
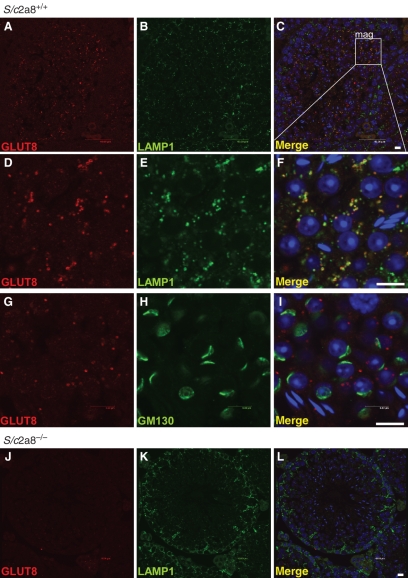
Co-localization of GLUT8 with LAMP1 in mouse testis. Immunohistochemistry of paraffin-embedded testis sections from wild-type (A–I) and GLUT8-deficient mice (*Slc2a8*^*−/−*^) (J–L). GLUT8 was not detectable in testis from GLUT8 knockout animals (J). In testis from wild-type animals (*Slc2a8*^+/+^), GLUT8 staining (A,D) overlaps (C,F) with the lysosomal protein LAMP1 (B,E). In contrast, the Golgi marker GM130 (H) did not co-localize with GLUT8 (G), as seen by the lack of overlap between the two proteins (I). Scale bars = 10 μm.

In order to identify the subcellular localization of endogenous GLUT8, we performed co-localization studies with markers of various intracellular compartments, using fluorescence labelling and confocal microscopy. Immunohistochemistry of GLUT8 in tissues such as testis or brain has been performed previously, but inconsistent results were obtained with regard to its subcellular localization [12–14,21,22]. In order to verify the specificity of the GLUT8 antibody, we used testis sections from GLUT8 knockout mice that have previously been shown to represent appropriate controls for this antiserum in conventional 3,3′-diaminobenzidine-based immunohistochemistry [23]. In addition, absence of the protein in mouse testis from GLUT8 knockout mice was demonstrated by western blot analysis of extracts of total membrane (Fig. S1A). As shown in Fig. 1, GLUT8 co-localizes with LAMP1, as indicated by the yellow punctured structures in the merged picture (Fig. 1C,F). A similar co-localization was observed for GLUT8 and the lysosomal protein LAMP2 (Fig. S1B). In contrast, the *cis*-Golgi marker GM130 did not show any overlap with GLUT8 staining (Fig. 1G–I). The specificity for fluorescent labelling of GLUT8 was demonstrated in testis sections from *Slc2a8*^*−/−*^ mice lacking GLUT8 (Fig. 1J–L).

### The N-terminus of GLUT8 interacts with endogenous, native AP1 and AP2

**Fig. 2:**
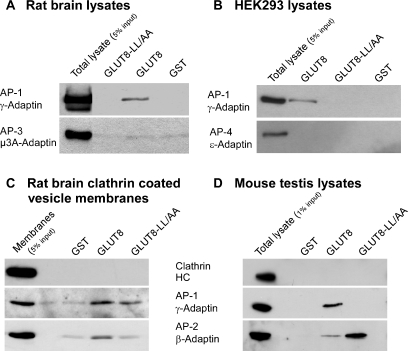
The [DE]XXXL[LI] motif of GLUT8 interacts with endogenous AP1 and AP2 in GST pulldown assays. GST pulldown assays were performed using lysates of rat brain (A) and HEK293 cells (B), clathrin-coated vesicle membranes enriched from rat brains (C), and lysates from mouse testis (D). The recombinant wild-type or mutated N-terminus of GLUT8 fused to GST was used as bait. The first lane in each panel represents a control for the lysates or membranes used in the pulldown assays (percentage of the total in parentheses).

Previous studies indicated that the adaptor complexes AP1 and AP2 interact with the dileucine motif of GLUT8 [5,19,20]. However, in these studies, GST pulldown assays were performed using recombinant AP subunits that yielded conflicting results with regard to the AP subunits that interact with the [DE]XXXL[LI] motif. In order to re-investigate this issue, we performed GST pulldown experiments using the N-terminal intracellular domain (NICD) of GLUT8 fused to GST. To date, the interaction of GLUT8 with AP3 or AP4 has not been addressed. AP3 mediates sorting of membrane proteins from endosomal compartments to late endosomes/lysosomes, and AP4 has been demonstrated to mediate direct sorting to lysosomes from the *trans-*Golgi network [18]. As GLUT8 is localized in a late endosomal/lysosomal compartment, a site where AP3- or AP4-mediated sorting might be required, we used GST pulldown experiments to investigate whether the NICD of GLUT8 interacts with AP3 or AP4. We incubated the immobilized fusion proteins with detergent-lysed rat brain homogenates (Fig. 2A), HEK293 cell extracts (Fig. 2B), clathrin-coated vesicle membranes isolated from porcine brains (Fig. 2C), and lysates from mouse testis (Fig. 2D) containing endogenous AP complexes. HEK293 cell lysates were required in order to test interaction with AP4, as commercially available antibodies react with the human AP4 protein only (ε-subunit). As shown in Fig. 2A,B, the GST–NICD fusion protein specifically binds to AP1, but not to AP3 or AP4 complexes. Mutation of the two adjacent leucines within the [DE]XXXL[LI] motif (LL/AA mutant) resulted in loss of AP1 binding, suggesting that an acidic cluster dileucine signal within the GLUT8 NICD is the major determinant for its association with AP1. Interaction of GLUT8 with recombinant AP2 has been reported previously [19,20]. As we were unable to detect binding to AP2 in cell homogenates (data not shown), we repeated the experiment using clathrin-coated proteins from brain and mouse testis lysates as a source of native AP1/AP2 complexes. Using these protein extracts, binding of both AP1 and AP2 to the GST–NICD fusion protein was readily detectable. However, mutation of the dileucine motif (LL/AA mutant) in GLUT8 did not completely abolish AP1/ and AP2/GLUT8 NICD interactions (Fig. 2C,D). This residual association with AP1 and AP2 might be due to high and variable concentrations of AP1 and AP2 in these extracts or could result from indirect binding of GLUT8 to AP complexes via unidentified tissue-specific bridging proteins. No specific interaction was observed with the GST control.

### Localization of GLUT8 is not altered in cells lacking AP3 subunits (*mocha* and *pearl* cells)

**Fig. 3:**
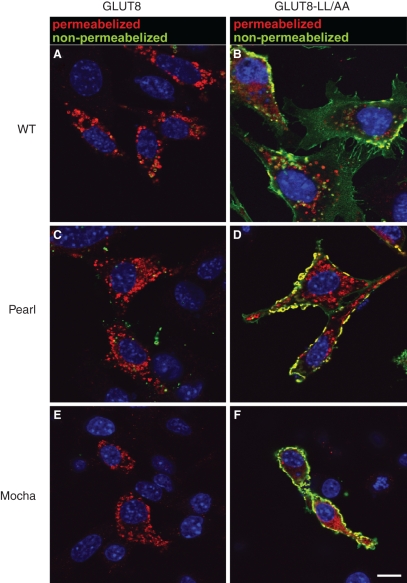
GLUT8 sorting is not altered in *mocha* and *pearl* cells lacking AP3 subunits. GLUT8 and the LL/AA mutant were overexpressed in either wild-type (WT) or AP3-deficient (*pearl*, *mocha*) mouse embryonic fibroblasts. Differential staining was performed in order to differentiate between plasma membrane and total GLUT8. Plasma membrane GLUT8 (A,C,E) or LL/AA mutant (B,D,F) was detected by incubating cells with the anti-haemagglutinin IgG in cell culture prior to fixation (in green). The haemagglutinin antibody recognizes plasma membrane GLUT8 via a haemagglutinin epitope that was introduced into the first extracellular loop of the transporter. Total GLUT8 was visualized using the C-terminal anti-GLUT8 IgG (in red) after fixation and permeabilization of cells. Scale bars = 10 μm.

**Fig. 4:**
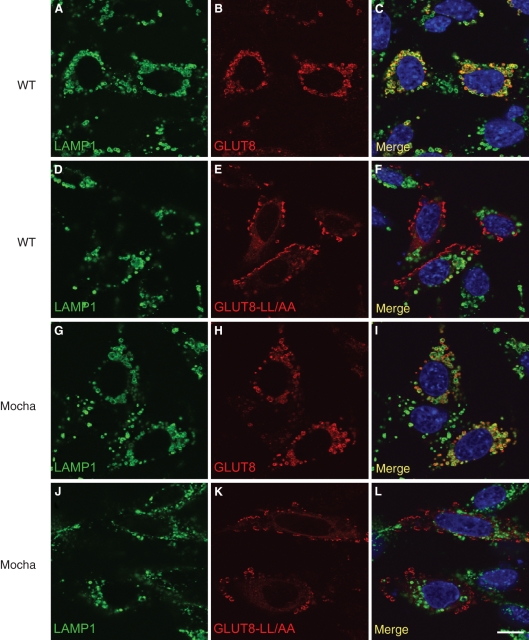
Co-localization of GLUT8 and LAMP1 is not affected in AP3-deficient cells. GLUT8 and the LL/AA mutant were overexpressed in either wild-type (A–F) or *mocha* (G–L) fibroblasts. Co-localization of GLUT8 and LAMP1 is seen to be independent of the presence (A–C) or absence (G–I) of AP3. However, the GLUT8-LL/AA mutant does not co-localize with LAMP1 (F,L), but instead appears at the plasma membrane in wild-type (E) as well as mutant (K) cells. Scale bars = 10 μm.

In order to confirm our biochemical data, we investigated the subcellular localization of GLUT8 in living cells. Given that sorting of several lysosomal proteins carrying a [DE]XXXL[LI] motif has been shown to involve AP3, we wished to determine whether AP3 is required for proper sorting of GLUT8, despite the fact that we were unable to detect an association between the proteins by GST pulldown assays. Mouse embryonic fibroblasts isolated from mice carrying mutations in AP3 subunits have already been widely used to study AP3-mediated sorting of lysosomal proteins [24]. We therefore analysed the localization of GLUT8 and the GLUT8-LL/AA mutant in cells that lack specific subunits of AP3. The mouse mutants *mocha* and *pearl* are deficient in the AP3 δ [25] and β3A [26] subunits, respectively. Failure to express one of the AP3 subunits leads to destabilization of the tetrameric complex and loss of AP3 functionality [24]. GLUT8 tagged within its extracellular loop with a haemagglutinin epitope [4] or the corresponding LL/AA mutant were overexpressed in wild-type, *mocha* or *pearl* fibroblasts (Fig. 3). By differential staining under non-permeabilizing or permeabilizing conditions, we found that GLUT8 was localized in intracellular punctae resembling late endosomes and lysosomes in all cell lines studied (Fig. 3A,C,E). By contrast, the LL/AA mutant was found predominantly at the cell surface (Fig. 3B,D,F). Consistent with these data, GLUT8 co-localized with LAMP1 in wild-type (Fig. 4A–C) and in AP3-deficient mutant cells (Fig. 4G–I). GLUT8-LL/AA did not show any detectable co-localization with LAMP1, and was found at the plasma membrane in all cell lines studied (Fig. 4D–F,J–L). Thus mutations leading to disruption of AP3 do not affect the steady-state distribution of GLUT8, nor do they affect its co-localization with the late endosomal/lysosomal marker protein LAMP1, a finding that is in agreement with our *in vitro* binding data.

### Targeting of GLUT8 in the absence of AP1 and AP2

**Fig. 5:**
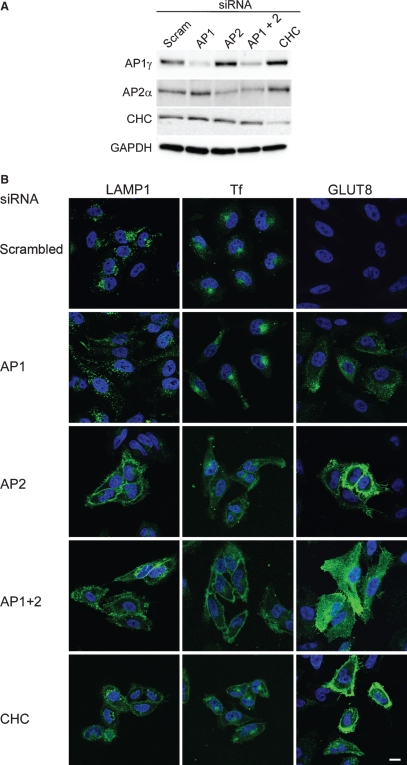
GLUT8 accumulates at the plasma membrane when cells are depleted of adaptor proteins or the clathrin heavy chain. (A) HeLa cells were transfected twice within 5 days with siRNA for AP1, AP2, AP1/AP2 or the clathrin heavy chain (CHC). After the second transfection, cells were analysed for efficient protein knockdown after 48 h by western blot analysis. (B) Alexa Fluor 488-conjugated transferrin uptake or LAMP1 antibody internalization were performed as described previously [40]. AP2 and CHC knockdown dramatically affects LAMP1 and transferrin receptor trafficking, leading to accumulation of the two proteins at the plasma membrane. Knockdown of AP1 leads to a modest level of GLUT8 in plasma membrane. In contrast, GLUT8 accumulates at the plasma membrane in cells transfected with AP2 or CHC siRNA. Scale bars = 10 μm.

In order to investigate the contribution of AP adaptors, most notably AP1 and AP2, to GLUT8 sorting we downregulated individual adaptor complex subunits or clathrin heavy chain (CHC) in Hela cells stably expressing GLUT8 by siRNA. Intracellular targeting of surface accessible GLUT8 was then assayed using an antibody feeding protocol. As shown in Fig. 5A, the siRNAs were capable of specifically downregulating their respective target AP subunits (Fig. 5A). 96 hours post-transfection of scrambled or target siRNAs Hela cells were exposed to antibodies directed against the haemagglutinin-tag of GLUT8, LAMP1 or to FITC-labeled transferrin. LAMP1 and LAMP2 both contain tyrosine based signals that bind to the μ subunits of AP adaptor complexes [45]. Sorting of LAMPs to lysosomes occurs directly from the TGN as well as via an indirect pathway involving clathrin/AP2 [40]. Cells transfected with scrambled siRNA displayed an unperturbed localization of internalized LAMP1 and TfR in distinct endosomal compartments. GLUT8 was not detectable by antibody feeding in this assay, suggesting that surface exposed pools of GLUT8 are very small under these conditions. Previous experiments demonstrated that plasma membrane GLUT8 can be detected that originates from the biosynthetic pathway traversing the plasma membrane [4]. Knockdown of AP1 caused a comparably minor re-distribution for LAMP1 to peripheral endosomal puncta (Fig. 5B) and led to a modest accumulation of GLUT8 at the plasma membrane (Fig. 5B). Knockdown of AP2 or AP1 and AP2 in combination resulted in a major redistribution of both LAMP1 and the TfR to the cell surface, reflecting the contribution of clathrin/AP2-mediated endocytosis to the sorting of both proteins. In line with this interpretation, a similar phenotype was observed following knockdown of clathrin (Fig. 5B). Strikingly, GLUT8 accumulated at the plasma membrane in cells depleted of either AP2 or clathrin (Fig. 5B). These data indicate that sorting of GLUT8 to lysosomes occurs via adaptor complex mediated mechanisms involving both AP2 and also AP1. This is also consistent with a previous report [20]. The current data examining GLUT8 sorting suggest that a fraction of GLUT8 traverses the plasma membrane, from where it is endocytosed via an AP2 and clathrin-dependent mechanism before being sorted to its final late endosomal/lysosomal destination.

### Co-localization of GLUT8 and LAMP1 in cells lacking adaptor proteins AP1, AP2 or CHC

If the above hypothesis is correct, one would also expect to detect alterations in the steady-state distribution of GLUT8 in siRNA-treated cells. We thus examined the effect of AP or clathrin downregulation on the localization of GLUT8 to LAMP1-positive late endosomes/lysosomes. GLUT8 and LAMP1 co-localized in cells treated with either control or target siRNAs (Fig. S2). However, differences were observed with regard to the intracellular distribution of LAMP1/GLUT8-containing organelles. Depletion of AP2 or clathrin resulted in a compact, perinuclear distribution of the organelles containing both proteins, whereas knockdown of AP1 had little effect. These data confirm the results obtained by antibody feeding of GLUT8, and suggest that clathrin/AP2-mediated endocytosis greatly contributes to the endosomal/lysosomal targeting of GLUT8 in HeLa cells.

### The N-terminal domain of GLUT8 contains a transplantable internalization signal

**Fig. 6:**
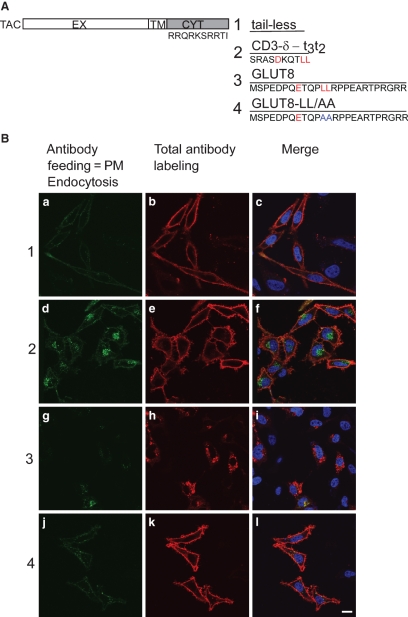
The [DE]XXXL[LI] motif of GLUT8 is sufficient for its intracellular retention. (A) Four chimeras (tailless interleukin-2 receptor α chain (TAC), a CD3-δ–TAC chimera, TAC–wild-type GLUT8 N-terminus and TAC–LL/AA-GLUT8 N-terminus) were transfected into HeLa cells. (B) Appearance of the proteins at the plasma membrane was assessed by TAC antibody internalization (labelled in green), and the overall distribution of the chimeric proteins was analysed after fixation and permeabilization of the cells (labelled in red). The tailless interleukin-2 receptor α chain construct appears at the plasma membrane only (B,a–c), whereas the CD3δt_3_t_2_–TAC chimera containing the EXXXLL consensus sequence is internalized from the membrane, as indicated by the internalized TAC antibody labelled in green (B,d). The GLUT8–TAC chimera is not targeted to the plasma membrane (green labelling in B,g). Mutating the dileucine motif of GLUT8 to LL/AA results in the opposite picture compared with the GLUT8–TAC protein, i.e. localization of the GLUT8-LL/AA–TAC chimera is restricted to the plasma membrane (B,k). Scale bars = 10 μm.

To determine the significance of the N-terminal dileucine signal in GLUT8 for its intracellular sorting, we constructed chimeric proteins comprising a truncated version of TAC (lacking its cytoplasmic tail) fused to various dileucine-based sorting motifs (Fig. 6A). TAC chimeras were overexpressed in HeLa cells, and their endocytosis was followed using an antibody internalization approach. The tailless TAC reporter protein lacking its cytoplasmic domain has been demonstrated to localize to the plasma membrane using a similar approach [27]. Fusion of the dileucine motif derived from the CD3 δ chain to tailless TAC was sufficient to target the chimera for internalization (Fig. 6B,d) as previously shown [27]. No plasmalemmal signal was detected for the corresponding GLUT8–TAC chimera (Fig. 6B,g) by either the antibody feeding approach (Fig. 6B,g) or antibody labelling by immunocytochemistry of the permeabilized cells (Fig. 6B,h). Instead, only intracellular GLUT8–TAC chimeric protein was detectable (Fig. 6B,h). This suggests that either internalization of this construct is too fast and efficient to be detected by this approach (similar to the antibody feeding in HeLa cells overexpressing GLUT8 and described above) and/or that its intracellular sorting occurs predominantly via a direct route from the *trans-*Golgi network, presumably involving AP1. In contrast, when the antibody feeding experiment was performed using with the LL/AA mutant GLUT8–TAC fusion protein, no endocytosed protein was labelled (Fig. 6B,j), while overall antibody staining detected the chimeric protein almost exclusively at the plasma membrane (Fig. 6B,k).

## Discussion

The present study demonstrates that endogenous GLUT8 localizes to a late endosomal/lysosomal compartment in spermatocytes and spermatids in the mouse testis. The [DE]XXXL[LI] sorting motif of GLUT8 interacts with AP1 and AP2 but not with AP3 or AP4. Furthermore, the [DE]XXXL[LI] motif represents a strong intracellular retention/sorting signal that is sufficient to target GLUT8 to its intracellular location, depending on its interaction with AP1 and/or AP2.

The physiological role of the evolutionarily ‘oldest’ class III GLUT family isoforms is not understood – especially in the context of their intracellular localization as described for all class III members [4,5,8,9,28]. This raises the question of whether these transporters are involved in intracellular substrate transport, or whether so far unknown conditions exist that result in a plasma membrane function for class III GLUTs.

Intracellular hexose transport has been shown to occur across lysosomal membranes [29,30], and has been postulated to occur in the endoplasmic reticulum [31,32]. Based on these data, it seems reasonable to speculate that transport of hexoses or other metabolites occurs across intracellular membranes. However, transporters accounting for glucose release from the endoplasmic reticulum [31] or export of sugars from lysosomes [32] have not yet been identified. The phenotype of GLUT8 knockout mice does not indicate a role for this transporter in embryo development as previously suggested [33,34] or in regulation of whole-body glucose homeostasis [23,32,35]. The results from two groups investigating the phenotype of *Slc2a8* null mice only show mild alterations in the metabolic profile of those animals, while indicating a significant physiological role for GLUT8 in the testis as well as in the brain, the tissues in which it is most abundant [23,32,35,36]. In order to obtain further insights into a possible functional role of GLUT8, we attempted to clarify its endogenous localization in the testis and to link those findings with a more in-depth characterization of the cell biology of the transporter. We were able to show for the first time that endogenous GLUT8 co-localizes with the late endosomal proteins LAMP1 and LAMP2. We provide clear evidence that, in the tissue in which it is most abundant, GLUT8 does not localize to the plasma membrane but is restricted in its localization to lysosome-related organelles. These data are in accordance with previous studies performed in cell lines showing a late endosomal/lysosomal localization for GLUT8. In addition, recent immunohistochemical findings demonstrated a diffuse cytoplasmic localization of the transporter in spermatids [15].

Based on a yeast two-hybrid assay and GST pulldown experiments, the dileucine motif of GLUT8 was indicated to interact with the β-subunits of AP1 and AP2 [20]. Although ‘tyrosine-based’ sorting signals conform to either the NPXY or YXXO consensus sequence and interact with AP1–4 via their μ subunits, the exact nature of AP interaction with dileucine signals of the [D/E]XXXL[L/I] motif is controversial. Recently, using yeast three-hybrid assays and GST pulldown experiments using recombinant AP subunits, various laboratories have shown that the [D/E]XXXL[L/I] motif interacts not only specifically but also selectively with hemicomplexes of AP1 γ/σ1, AP2 α/σ2 or AP3 δ/σ3 [19,37,38]. In addition, the N-terminus of GLUT8 has been shown to interact with hemicomplexes of AP1 γ/σ1 and AP2 α/σ2 [19]. More recently, X-ray crystallography provided a structural explanation of how a [D/E]XXXL[L/I] motif is recognized by AP2, and identified the σ2 subunit as the major site of interaction [39]. Rather than using recombinant AP subunits for GST pulldown experiments, we used native proteins to demonstrate that the [D/E]XXXL[L/I] motif of GLUT8 interacts with AP1 and AP2, but not with AP3 or AP4. Based on the late endosomal/lysosomal localization of GLUT8, we initially hypothesized that sorting of GLUT8 might involve interaction of its dileucine motif with AP3 and/or AP4. In addition to demonstrating that GLUT8 does not interact with AP3 or AP4, we showed that localization of the transporter is not altered in cells lacking AP3. Our findings are in accordance with other studies showing that the steady-state localization of lysosomal proteins is not significantly affected in cells lacking AP3 subunits [40].

The siRNA approach has been successfully used to analyse AP- or CHC-mediated sorting for LAMP1 and LAMP2 [40]. AP2 or CHC siRNA treatment in HeLa cells stably expressing GLUT8 resulted in accumulation of the protein in plasma membranes, whereas AP1 knockdown led to only a moderate alteration of its subcellular localization. We also demonstrated that knockdown of AP1 or AP2 affected the distribution of both GLUT8 and LAMP1. The effect of AP knockdown on LAMP1 localization observed here is in agreement with findings that elucidated the role of AP in sorting mechanisms of integral lysosomal membrane proteins [40]. It was shown that mainly AP2 and clathrin are required for efficient delivery of LAMPs to lysosomes, implying that a significant population of LAMPs traffic via the plasma membrane en route to lysosomes [40]. Our data suggest that sorting of GLUT8 shows similarities to that of LAMPs. At steady state, GLUT8 does not recycle, and is found to be exclusively associated with intracellular membranes. In addition, a biosynthetic pathway appears to exist that involves sorting of GLUT8 via the plasma membrane, as previously suggested [4].

Using the TAC chimera approach, we were able to demonstrate that the dileucine signal of GLUT8 is sufficient for its intracellular retention and represents a strong intracellular sorting signal. Our data are supported by a recent study that compared the [D/E]XXXL[L/I] sorting motifs between GLUT8 and GLUT12, showing that this sorting signal very specifically controls localization and sorting of both transporters [41]. The absence of the GLUT8–TAC chimera at the plasma membrane indicated that a majority of the chimeric protein is directly sorted to an intracellular compartment and/or that AP2-dependent endocytosis occurs very rapidly. Mutating the LL signal to AA in the TAC chimeric protein totally abolished sorting of the chimera to an intracellular location, and led to mis-routing to the plasma membrane and/or blocking of its endocytosis.

Although the physiological role of GLUT8 remains unknown, our data may provide a link between cell biological data and observations from phenotypical analysis of GLUT8 knockout mice. GLUT8 may be involved in intracellular transport of metabolites thereby secondarily affecting ATP concentrations and mitochondrial function as observed in GLUT8 deficient sperm cells [42]. Therefore, future studies require identification of other substrates of GLUT8 in order to clarify the intracellular function of the transporter [42].

## Experimental procedures

### DNA constructs, plasmids and antibodies

The mouse GLUT8 wild-type or LL/AA mutant cloned into a mammalian expression vector (pcDNA3) has been described previously [4,5]. A GLUT8 antibody was raised against two peptide epitopes, and was previously shown to recognize GLUT8 by immunohistochemistry [23]. A second GLUT8 antibody that was raised in rats against an epitope located in the N-terminus of GLUT8 was used to detect the protein by western blot analysis. The following antibodies were used in the present study: monoclonal mouse anti-haemagglutinin tag (Covance Research Products Inc., Berkeley, CA, USA), anti-mouse Golgi marker GM130 and anti-mouse LAMP1 (1D4B), mouse anti-AP1γ, -AP2α, -AP3δ and -AP4ε, mouse anti-clathrin heavy chain (BD Pharmingen, Heidelberg, Germany), mouse monoclonal anti-human LAMP1 (H4A3), mouse monoclonal anti-human LAMP2 (H4B4), and rat anti-mouse LAMP2 (ABL-93) (Developmental Studies Hybridoma Bank at the University of Iowa). The mouse anti-interleukin-2 receptor α chain (anti-TAC) was a kind gift from R. Kroczek (Robert Koch Institute, Berlin, Germany). Fluorescent and horseradish peroxidase-conjugated secondary antibodies were purchased from Invitrogen (Karlsruhe, Germany) and Dianova (Hamburg, Germany), respectively.

### Western blot analysis of total membranes from testis of wild-type and GLUT8 knockout mice

Total membranes were prepared as previously described [4]. Western blot analysis was performed with 20 μg of total membranes, and GLUT8 was detected using an antibody raised in rats against the N-terminus of GLUT8 (residues MSPEDPQETQPLLRPC). After protein transfer, nitrocellulose membranes were exposed to GLUT8 antiserum (10 μg·mL^−1^) overnight at 4 °C. Membranes were subsequently probed with a horseradish peroxidise-conjugated goat anti-rat IgG (Thermo Scientific Pierce, Rockford, IL, USA), and developed by enhanced chemiluminescence (Amersham Pharmacia Biotech, Freiburg, Germany). Images were taken using a Fujifilm LAS-1000 camera and processed using image reader las-1000 software (Fujifilm Germany, Düssdeldorf; Germany).

### Immunohistochemistry on mouse testis sections and immunocytochemistry

Paraffin-embedded mouse testis sections were used for labelling of GLUT8 by immunohistochemistry with the previously described antiserum [23]. Rehydrated paraffin sections were treated with citrate buffer (target retrieval solution, ChemMate™, Dako Cytomation, Hamburg, Germany), and primary antibodies were applied overnight at 4 °C in antibody dilution medium (antibody diluent with background reducing components, Dako Cytomation). GLUT8 was labelled with a biotin-conjugated anti-rabbit IgG secondary antibody (1 : 800) and visualized using Alexa Fluor 546-labelled streptavidin (Invitrogen). For co-localization experiments, the Golgi apparatus or lysosomes were labelled with anti-GM130 IgG or anti-LAMP1 and anti-LAMP2 IgGs, respectively, at dilutions of 1 : 100. For secondary detection, Alexa Fluor 488-conjugated goat anti-mouse IgG (GM130, LAMP1) or donkey anti-rat IgG antibody (LAMP2) was used. The immunocytochemistry method has been described previously [4]. Nuclei were stained using TOPRO-3 iodide (Invitrogen), and samples were mounted in Vectashield (Vector Labs, Burlingame, CA, USA). Images were obtained using a Leica LCS confocal laser scanning microscope (Leica, Wetzlar, Germany).

### Cell culture and transfections

HeLa cells maintained in Dulbecco’s modified Eagle’s medium supplemented with 10% fetal bovine serum, 1% penicillin/streptomycin and sodium pyruvate were transfected with Lipofectamine 2000 (Invitrogen). Cells stably expressing the transgene were obtained (0.8 mg·mL^−1^ G418), and clonal selection was achieved by limited dilution. Mouse embryonic fibroblasts lacking either the δ (*mocha*) or the β3A (*pearl*) subunits of the adaptor protein AP3 were a kind gift from Stefan Höning (Institute for Biochemistry, University of Cologne, Germany). The cells were grown in Dulbecco’s modified Eagle’s medium supplemented with 10% fetal bovine serum and 1% penicillin/streptomycin. Transfection of fibroblasts with GLUT8 or GLUT8-LL/AA plasmids was performed using Lipofectamine 2000 (Invitrogen).

### Adaptor protein and clathrin chain knockdown by siRNA

Previously described siRNAs [40] were used to achieve knockdown of human AP subunits and the clathrin heavy chain (CHC): CHC, 5′-AUCCAAUUCGAAGACCAAU(dTdT)-3′; AP1γ, 5′-GUUCCUGAACUUAUGGAGA(dTdT)-3′; AP2μ, 5′-GUGGAUGCCUUUCGGGUCA(dTdT)-3′; scrambled siRNA, 5′-GUAACUGUCGGCUCGUGGU(dTdT)-3′.

HeLa cells stably expressing GLUT8 were transfected twice within 5 days with the corresponding siRNA using oligofectamine (Invitrogen).

### GST pulldown assays

Plasmids containing the wild-type or mutated (LL→AA) N-terminus of GLUT8 cloned in-frame into the GST fusion vector pGEX3X were provided by H. Al-Hasani (Department of Pharmacology, German Institute of Human Nutrition, Potsdam Rehbruecke, Nuthetal, Germany) [20]. The first 33 amino acids of stonin 1 containing a WXXF motif that interacts with AP2 as demonstrated by Walther *et al.* [43] was used as a positive control for pulldown assays. Protein expression was induced by addition of 0.5 mm isopropyl thio-β-d-galactoside for 2 h at 37 °C. After 4 h, cells were lysed by sonication (60 s at 60% power), with addition of lysozyme (1 mg·mL^−1^) and 1% Triton X-100. A clear supernatant was obtained after centrifugation for 30 min at 33 000 ***g***. GST beads (80 μL) were added and mixed for 2 h. Beads were washed three times with 10 mL NaCl/P_i_ and 100 mm NaCl for 10 min each and finally once in NaCl/P_i_.

Pulldown experiments were performed using rat brain extracts, HEK293 cell lysates, clathrin-coated vesicles enriched from rat brains, and lysates prepared from mouse testis. Rat brains were homogenized in 10 mL of buffer (20 mm Hepes, 100 mm NaCl, 1 mm MgCl_2_ and 1 mm phenylmethanesulfonyl fluoride) using a Teflon® homogenizer with 15 strokes at 900 rpm. A postnuclear supernatant was obtained by centrifugation at 1000 ***g*** for 10 min, and Triton X-100 was added to the lysate to a final concentration of 1%. The lysate was kept on ice for 10 min with occasional vortexing. Lysates were cleared by centrifugation at 36 000 ***g*** for 15 min and at 20 000 ***g*** for 20 min. The supernatant was recovered and used at a concentration of 3 mg protein·mL^−1^.

Extracts from mice testis (total six) were prepared by homogenization in 3 mL 30 mm Hepes pH 7.4, 50 mm KCl, 2 mm MgCl_2_, phenylmethanesulfonyl fluoride and protease inhibitor cocktail, plus 1% Triton X-100 in a Teflon® homogenizer (12 strokes at 1100 rpm). After lysis on ice for 30 min, protein lysates were obtained from the supernatant after centrifugation at 14 000 ***g*** for 20 min and at 65 000 ***g*** for 15 min (protein concentration 11 mg·mL^−1^). The pulldown was performed using 200 μg of GST fusion proteins and 0.75 mL protein extract by incubation for 3 h with end-over-end rotation. The samples were washed four times in homogenization buffer and once in the same buffer without detergent. Proteins were eluted from the beads twice with a total of 90 μL SDS–PAGE sample buffer. One third of each sample was analysed by western blot analysis.

In the assays for interaction of GST constructs with AP4, HEK293 cell lysates were used, as the AP4 antibody only recognizes the human protein (ε-subunit). HEK293 cell lysates were obtained by homogenization in a Teflon® homogenizer in 20 mm Hepes, 100 mm NaCl, 1 mm MgCl_2_ and 1 mm phenylmethanesulfonyl fluoride (20 strokes at 2000 rpm). After addition of 1% Triton X-100, cell homogenates were kept on ice for 20 min and centrifuged at 13 000 ***g*** for 20 min. The supernatant was recovered and used at a concentration of 4 mg·mL^−1^ protein. Pulldown assays were performed using 1 mL of protein lysate in 20 mm Hepes, 100 mm NaCl, 1 mm MgCl_2_, 1 mm phenylmethanesulfonyl fluoride and 1% Triton X-100. Fusion protein was added (100 μg), and samples were kept on a rotating wheel for 2 h. Samples were washed three times briefly with 1 mL of buffer. After the final wash in buffer without Triton X-100, proteins were eluted from the beads using 50 μL SDS–PAGE sample buffer, and boiled for 5 min. Samples were separated by 10% SDS–PAGE, and western blot analysis was performed. Coated vesicles were isolated from rat brains using the procedure described by Maycox *et al.* [44]. For pulldown assays, 75 μg coated vesicles were incubated with 40 μg GST fusion protein under the conditions described for mice testis extracts.

### Construction of chimera, transfection and anti-TAC internalization assay

Chimeras consisting of GLUT8 or GLUT8-LL/AA N-terminus and the external and transmembrane domain of the human TAC antigen (interleukin-2 receptor α chain) were constructed based on a tailless TAC construct (without the cytoplasmic domain) (Fig. 6A). As positive control for a dileucine-based lysosomal targeting motif, a chimeric protein (TTγt_3_-t_2_) was used, consisting of the γ subunit of the T-cell antigen receptor fused to the TAC antigen [27]. The γ subunit of CD3 has been extensively studied, and the TAC chimera approach has been proven to be a valuable tool to study sorting motifs [27,45,46]. HeLa cells were transiently transfected with the TAC tailless, TACγt_3_-t_2_, TAC–GLUT8 and TAC–GLUT8-LL/AA constructs using Lipofectamine 2000 (Invitrogen). Two days after transfection, cells were labelled with anti-TAC IgG (1 : 1000 diluted in Opti-MEM; Invitrogen, Karlsruhe, Germany) for 30 min at 4 °C. After one change of medium (to Opti-MEM at 37 °C), plasma membrane antigens were allowed to internalize for 30 min at 37 °C. The cells were then fixed with 3% paraformaldehyde (Sigma-Aldrich, Seelze, Germany) for 10 min on ice, and surface-bound TAC antibody was blocked using goat anti-mouse serum [goat anti-mouse IgG from Dianova (Hamburg, Germany) at a 1 : 5 dilution in goat serum dilution buffer, consisting of 30% normal goat serum, 450 mm NaCl in NaCl/P_i_ pH 7.4] for 2 h at room temperature. Cells were permeabilized and blocked with goat serum dilution buffer containing 0.2% saponin for 10 min. For detection of internalized TAC antibody, a goat anti-mouse Alexa Fluor 488-conjugated IgG (Invitrogen) was added for 1 h. Cells were then washed three times for 10 min each with NaCl/P_i_/0.02% saponin. For total TAC staining (intracellular and cell surface), the specimens were incubated for 1 h with the TAC antibody diluted 1 : 1000 as described above. As secondary antibody, an Alexa Fluor 546-conjugated goat anti-mouse IgG was added for 30 min, and nuclei were stained using TOPRO-3 iodide. Cells were washed, and cover slips were mounted in Vectashield (Vector Labs). Specimens were examined using a Leica LCS confocal laser scanning microscope in sequential scanning mode.

## Abbreviations

AP: adaptor protein
CHC: clathrin heavy chain
GLUT: glucose transporter protein family
GST: glutathione *S*-transferase
NICD: N-terminal intracellular domain
TAC: lumenal domain of the IL-2 receptor alpha chain

## Supporting information

The following supplementary material is available:

**Fig. S1.** GLUT8 protein is (A) not detected by western blot analysis in testis of GLUT8 knockout mice, and (B) co-localizes with the lysosomal protein LAMP2 in testis of wild-type mice.

**Fig. S2.** GLUT8 is co-localized with LAMP1 in cells treated with siRNA targeting AP1, AP2 or CHC.

This supplementary material can be found in the online version of this article.

Please note: As a service to our authors and readers, this journal provides supporting information supplied by the authors. Such materials are peer-reviewed and may be re-organized for online delivery, but are not copy-edited or typeset. Technical support issues arising from supporting information (other than missing files) should be addressed to the authors.
