# Supplementary figures and images for: Lysosomal localization of GLUT8 in the testis – the EXXXLL motif of GLUT8 is sufficient for its intracellular sorting via AP1- and AP2-mediated interaction
Source: FEBS J. 2009 Jul;276(14):3729–43. doi: 10.1111/j.1742-4658.2009.07089.x (PMC2730553; doi:10.1111/j.1742-4658.2009.07089.x)

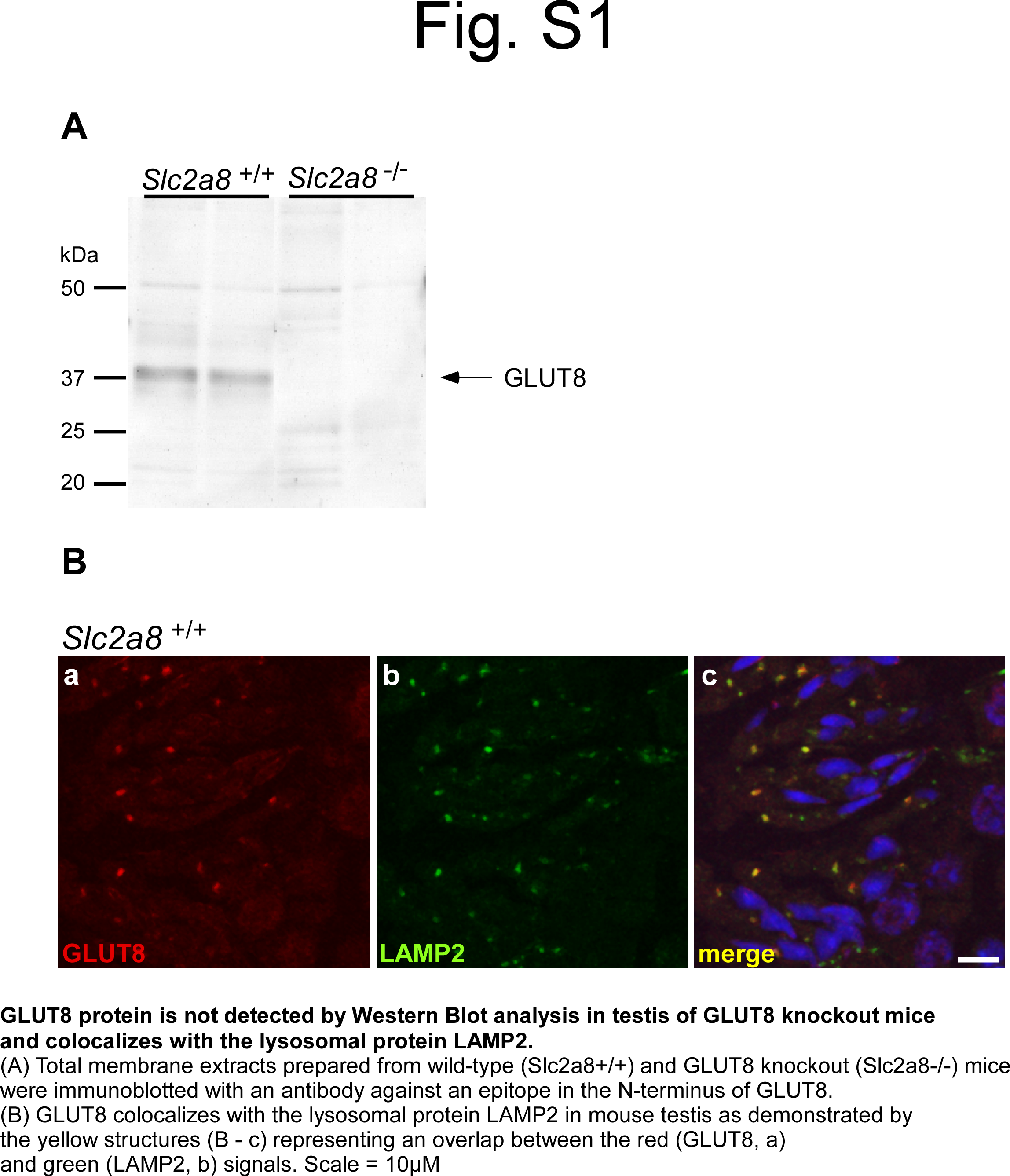

Supplement: Supplementary file 1 [file ejb0276-3729-SD1.tif]

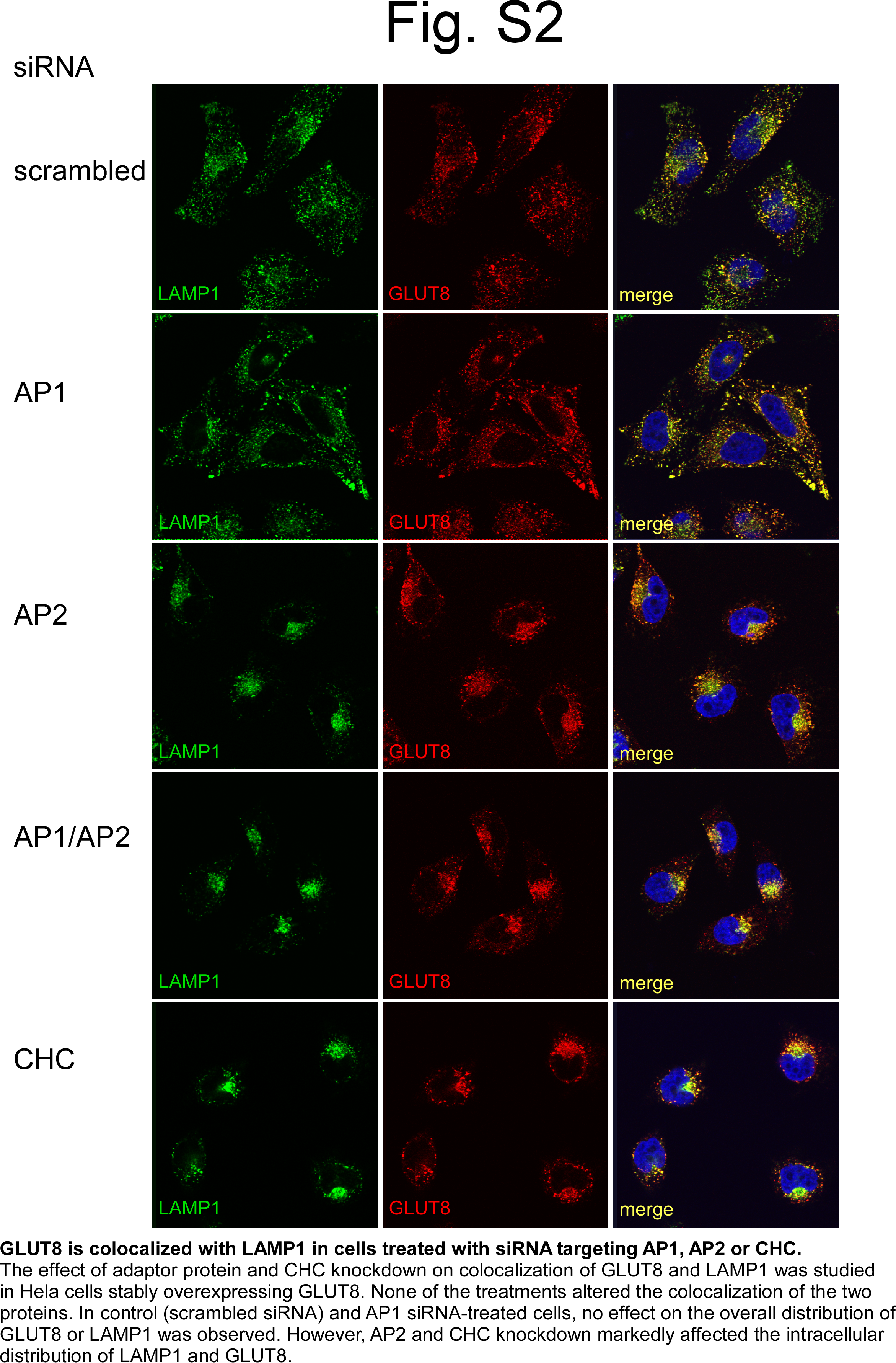

Supplement: Supplementary file 2 [file ejb0276-3729-SD2.tif]
